# Caffeic acid phenethyl ester suppresses metastasis of breast cancer cells by inactivating FGFR1 via MD2

**DOI:** 10.1371/journal.pone.0289031

**Published:** 2023-07-25

**Authors:** Qilu Fang, Wenxiu Xin, Liangsheng Chen, Yuxuan Fu, Yajun Qi, Haiying Ding, Luo Fang

**Affiliations:** 1 Zhejiang Cancer Hospital, Hangzhou Institute Medicine (HIM), Chinese Academy of Sciences, Hangzhou, Zhejiang, China; 2 Postgraduate Training Base of Zhejiang Cancer Hospital, Wenzhou Medical University, Wenzhou, Zhejiang, China; Xiangya Hospital Central South University, CHINA

## Abstract

**Background:**

Tumor metastasis is the main cause of death for breast cancer patients. Caffeic acid phenethyl ester (CAPE) has strong anti-tumor effects with very low toxicity and may be a potential candidate drug. However, the anti-metastatic effect and molecular mechanism of CAPE on breast cancer need more research.

**Methods:**

MCF-7 and MDA-MB-231 breast cancer cells were used here. Wound healing and Transwell assay were used for migration and invasion detection. Western blot and RT-qPCR were carried out for the epithelial-to-myofibroblast transformation (EMT) process investigation. Western blot and immunofluorescence were performed for fibroblast growth factor receptor1 (FGFR1) phosphorylation and nuclear transfer detection. Co-immunoprecipitation was used for the FGFR1/myeloid differentiation protein2 (MD2) complex investigation.

**Results:**

Our results suggested that CAPE blocks the migration, invasion, and EMT process of breast cancer cells. Mechanistically, CAPE inhibits FGFR1 phosphorylation and nuclear transfer while overexpression of FGFR1 reduces the anti-metastasis effect of CAPE. Further, we found that FGFR1 is bound to MD2, and silencing MD2 inhibits FGFR1 phosphorylation and nuclear transfer as well as cell migration and invasion.

**Conclusion:**

This study illustrated that CAPE restrained FGFR1 activation and nuclear transfer through MD2/FGFR1 complex inhibition and showed good inhibitory effects on the metastasis of breast cancer cells.

## Introduction

Breast cancer is still one of the most prevalent tumors in the world, accounting for 25% of all new female cancers. As of 2018, there were an estimated 2,088,849 cases of female breast cancer worldwide [1]. Breast cancer has strong recurrence and metastasis characteristics, which make clinical treatment difficult. It is currently known as a significant "killer" of cancer deaths in women, accounting for 15% of female cancer deaths [2].

Caffeic acid phenethyl ester (CAPE) is a natural phenolic compound extracted from propolis and has a variety of biological properties, including anti-inflammatory, antioxidant, anti-virus, immune regulation, and anti-tumor [3–5]. Myeloid differentiation protein2 (MD2)/toll-like receptor4 (TLR4) is considered as a membrane target of CAPE, and data shows that CAPE can bind to the key hydrogen bond and hydrophobic region of MD2, weakening the activation of downstream signals [6,7]. CAPE has excellent anti-tumor effects in multiple studies; notably, it has no toxicity to normal cells, which is an important advantage to becoming an anti-tumor drug [8–10]. Fibroblast growth factor receptor 1 (FGFR1) is a member of receptor tyrosine kinase (RTK) family and can play an important role in the carcinogenesis and tumor metastasis of breast cancer [11]. Studies have shown that FGFR1 could be activated and transplanted into the nucleus. Nucleus FGFR1 could interact with chromatin and regulate gene transcription to promote cancer progression [12,13].

In this study, we found that CAPE inhibits migration, invasion, and EMT of breast cancer cells through FGFR1 inactivation, including FGFR1 phosphorylation and nuclear transfer. We further found MD2 can combine with FGFR1, and CAPE mediates FGFR1 inactivation via MD2. Our results identify CAPE as an effective drug for metastatic breast cancer with a potential new mechanism.

## Materials and methods

### Reagents

CAPE was purchased from Selleckchem (Shanghai, China). Dimethyl sulfoxide (DMSO) was purchased from Sigma Aldrich (St. Louis, USA). Primary antibodies: anti-Vimentin (cat. 60330-1-Ig), anti-E-cadherin (cat. 20874-1-AP), anti-Occludin (cat. 27260-1-AP), anti-Laminb (cat. 12987-1-AP) and anti-GAPDH (cat. 60004-1-Ig) were obtained from Proteintech (Wuhan, China). Anti-p-FGFR1(Tyr653/654) (#52928) and anti-FGFR1 (#9740) were obtained from Cell Signaling Technology (Shanghai, China). Anti-MD2 (ab24182) was obtained from Abcam (Shanghai, China). Anti-TLR4 (sc-293072) was obtained from Santa Cruz Biotechnologylnc (Shanghai, China). Fetal bovine serum (FBS) and RPIM 1640 medium were purchased from Gibco (Paisley, UK). Secondary antibodies: HRP-conjugated Affinipure Goat Anti-Mouse IgG (cat. no. SA00001-2) and HRP-conjugated Affinipure Goat Anti-Rabbit IgG (cat. no. SA00001-2) were purchased from Proteintech (Wuhan, China). Lipofectamine 2000 were obtained from Invitrogen (Grand Island, USA). Trizol, SYBR Green qPCR kit and reverse transcription kit was obtained from TaKaRa (Tokyo, Japan). All other reagents were purchased from Beyotime (Shanghai, China).

### Cell culture

Human breast cancer cell lines MCF-7 and MDA-MB-231 were obtained from the National Collection of Authenticated Cell Cultures (Shanghai, China). Cells were cultured in RPMI-1640 medium with 10% FBS, 1% penicillin, and streptomycin solutions in a 37°C, 5% CO_2_ incubator.

### Cell transfection

Cells were transfected with small interfering RNA targeting MD2 or human FGFR1 plasmid (pWZL_Neo_Myr_Flag_FGFR1) purchased from Addgene (Cat#20486) using Lipofectamine 2000 and transfection medium, and then changed with fresh medium after 6 h. The siRNA sequences of MD2 are listed in S1 Table and sequence 1 is used in the next experiments.

### Wound healing assay

Cells were spread in 12-well plates and began assaying when they reached 80%-90% confluence. Cells cultured in serum-free medium were scratched and photographed at 0 h. After incubating with CAPE for 24h and 48h or transfected with siRNA of MD2 for 24h or FGFR1 plasmid for 12h, the cells were respectively photographed again. The wound was monitored using light microscopy (Nikon, Tokyo, Japan).

### Transwell assay

Cells were plated in the upper of matrigel-coated 24-well transwell chambers (Corning, New York, USA) and cultured with the serum-free medium while the lower chamber was with medium containing 10% FBS. After incubating with CAPE for 24h or transfected with siRNA of MD2 for 24h or FGFR1 plasmid for 12h, noninvasive cells on the upper surface were removed with cotton-tipped swabs while invasive cells on the lower surface of the membrane were stained with crystal violet. Images were captured with light microscopy (Nikon, Tokyo, Japan).

### Western blot assay

Cells were seeded in 6-well plates and treated with CAPE or transfected with siRNA of MD2 or FGFR1 plasmid. Then the treated cells were lysed with PIPA lysis buffer (Beyotime, Shanghai, China) and the protein concentration was detected using a BCA protein assay kit (Beyotime, Shanghai, China). Samples were separated by 10% SDS-PAGE and transferred to PVDF membranes. The membrane was blocked with 5% nonfat dry milk at room temperature for 1.5 h and incubated with the primer antibody at 4°C overnight. Following, the membranes were incubated with anti-Mouse or anti-Rabbit secondary antibodies, and visualized using enhanced chemiluminescence reagents (Beyotime, Shanghai, China). Protein signals were analyzed using ChemiDoc XRS+ software (Bio-Rad, USA).

### Real time quantitative PCR (RT-qPCR) assay

Cells were seeded in 6-well plates and treated with CAPE. Total RNA extracted from the cell plates was performed by Trizol reagent and reverse transcribed into cDNA with a two-step reverse transcription kit. The quantitative PCR was carried out using the SYBR Green qPCR kit by LightCycle* 480 II (Roche Diagnostic Canada, QC). PCR results were normalized to β-actin expression. The primer sequences of Vimentin, E-cadherin, Occludin, and β-actin are listed in S2 Table.

### Immunofluorescence assay

Cells were crawled in 6-well plates and treated with CAPE or transfected with siRNA of MD2. Cells were fixed with cold methanol for 10 min, blocked with 1% BSA (Beyotime, Shanghai, China) for 30 min at 37°C, and then incubated with anti-FGFR1 antibody (1:300) overnight at 4°C. Following, the cells were incubated with DyLight488 goat anti-rabbit secondary antibody (1:500, Beyotime, Shanghai, China) for 1 h at room temperature. DAPI (Beyotime, Shanghai, China) was used for nuclei staining. Slides were viewed with a fluorescence microscope (Nikon, Tokyo, Japan).

### Co-immunoprecipitation assay

Total lysates extracted from cells were immunoprecipitated with anti-FGFR1 or IgG overnight at 4°C. Then precipitated with protein A/G-agarose beads for 2 h at 4°C (Beyotime, Shanghai, China). Next, beads were washed with PBS 5 times and prepared into protein samples analyzed by western blotting assay.

### Immunohistochemical data

FGFR1 immunohistochemical data of breast cancer tissues were obtained from the PATHOLOGY of the human protein alts database (*https://www.proteinatlas.org*). The patient id was 1910 and 2805.

### Statistical analysis

All experiments were performed at least in triplicates. Data were analyzed using the Prism 5 Program (Graph Pad, San Diego, CA, USA). Values are presented with individual data points + mean. Statistical difference was determined by one-way ANOVA followed by Tukey’s range test. A P-value of <0.05 was regarded statistically significant.

## Results

### CAPE significantly inhibited cell migration and invasion

**Fig 1:**
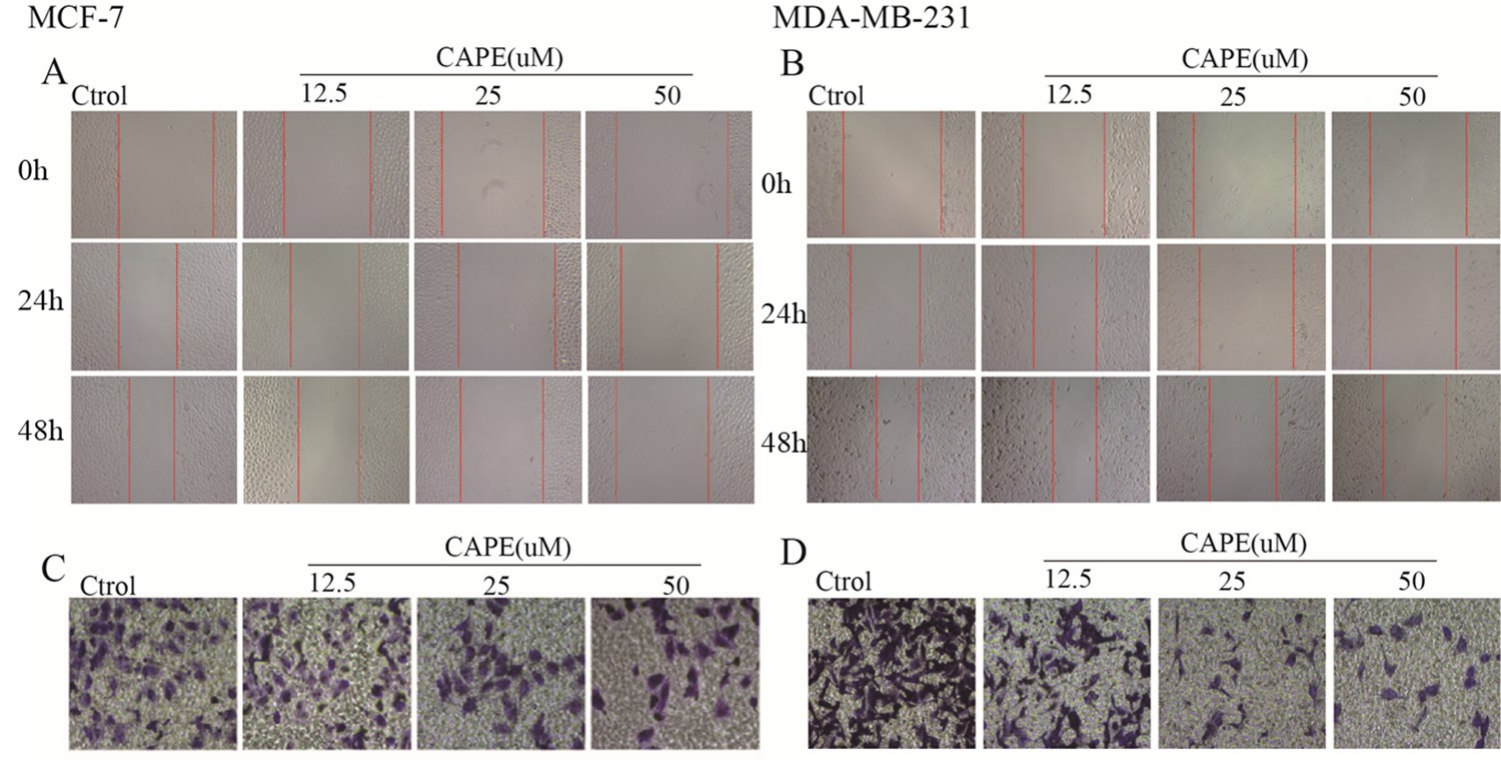
CAPE inhibited the migration and invasion of MCF-7 and MDA-MB-231 cells. Cells treated with or without CAPE for 24 h or 48 h, (**A-B**) wound healing was used for migration evaluation (100x) and (**C-D**) Transwell assay was conducted for invasion evaluation (200x). Experiments were repeated for 3 times.

To observe the effect of CAPE on cell migration and invasion, MCF-7 and MDA-MB-231 cells were treated with CAPE at 12.5, 25, and 50 μM and then assessed by wound healing and transwell. We observed the motility of cells in the scratch area and found that CAPE significantly delayed cell migration (Fig 1A and 1C). The gap width at 24 h or 48 h standardized by 0 h was larger in the CAPE-treated groups when compared with control groups and presented a dose-dependent trend. Results showed the same in the cell invasion experiment, CAPE markedly reduced cell invasion ability (Fig 1B and 1D). Crystal violet staining showed that the number of invasion cell decreased after treatment with CAPE for 24 h. These results suggested that the migration and invasion of MCF-7 and MDA-MB-231 cells could be significantly inhibited by CAPE.

### CAPE obstructed epithelial-mesenchymal transition

**Fig 2:**
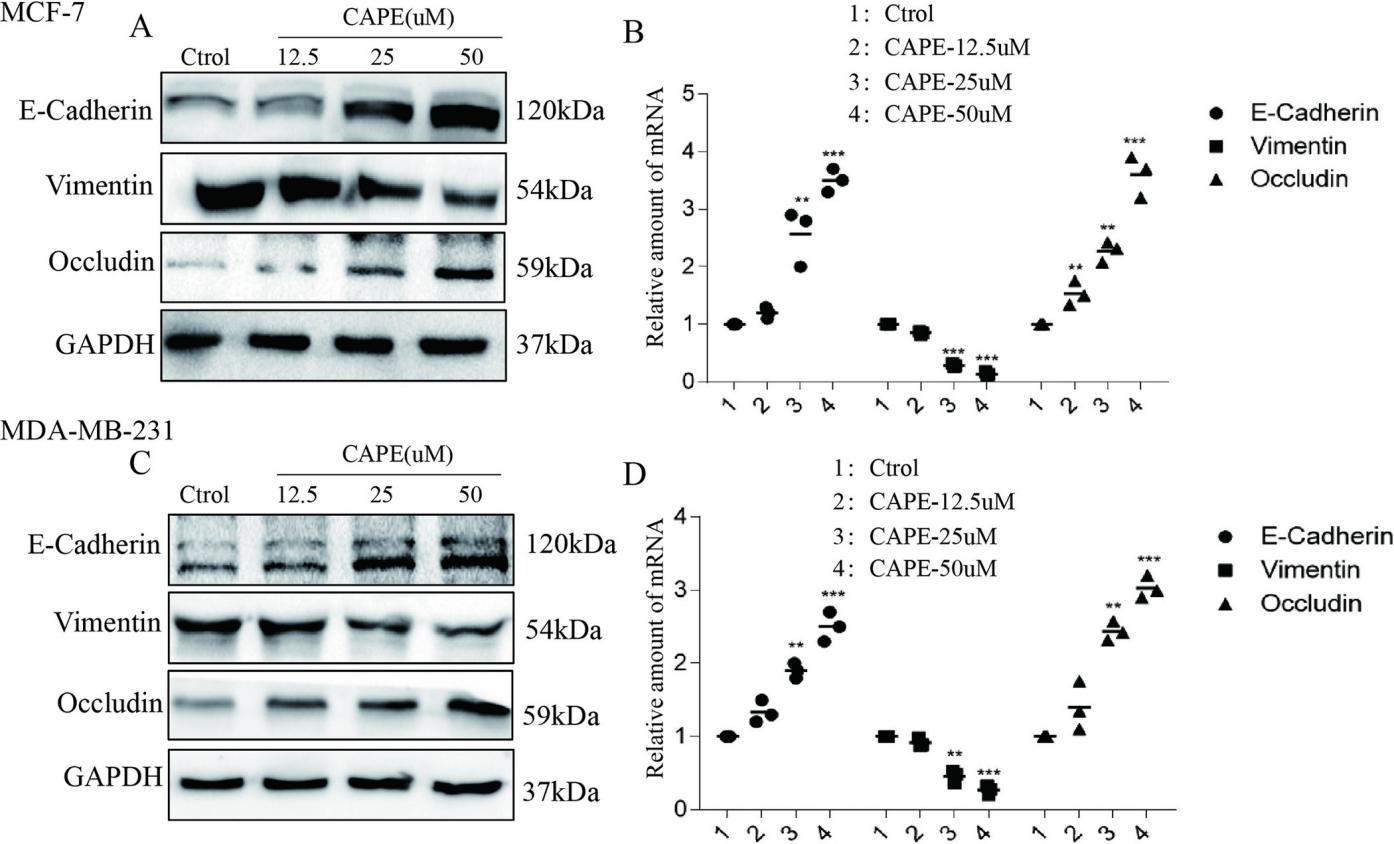
CAPE obstructed the epithelial-mesenchymal transition of MCF-7 and MDA-MB-231 cells. Cells were incubated with or without CAPE for 24 h or 12 h, western blot (**A, C**) and RT-qPCR (**B, D**) were performed for E-cadherin, Vimentin and Occludin detection. Experiments were repeated for 3 times. Values represent the mean (MCF-7: p = 0.0384, p = 0.0076 for E-Cadherin, p = 0.0081, p = 0.0056 for Vimentin, p = 0.0424, p = 0.0246, p = 0.0028 for Occludin, MDA-MB-231: p = 0.0431, p = 0.0085 for E-Cadherin, p = 0.0412, p = 0.0092 for Vimentin, p = 0.0324, p = 0.0066 for Occludin) *p<0.05, **p<0.01, ***p<0.001: CAPE-treated group compared control group.

To explore whether CAPE play a resistance role on cell EMT, MCF-7 and MDA-MB-231 cells were incubated with CAPE at 12.5, 25 and 50 μM for 24 h or 12 h. Western blot and RT-qPCR showed that CAPE-treated groups had a significantly change in expression of EMT related indicators compared to control group (Fig 2A–2D). CAPE treatment down regulated the expression of Vimentin and up regulated the expression of E-cadherin and Occludin both in protein and mRNA levels. These data indicated that CAPE suppressed cell epithelial-mesenchymal transition.

### CAPE intercepted FGFR1 activation and nuclear transfer

**Fig 3:**
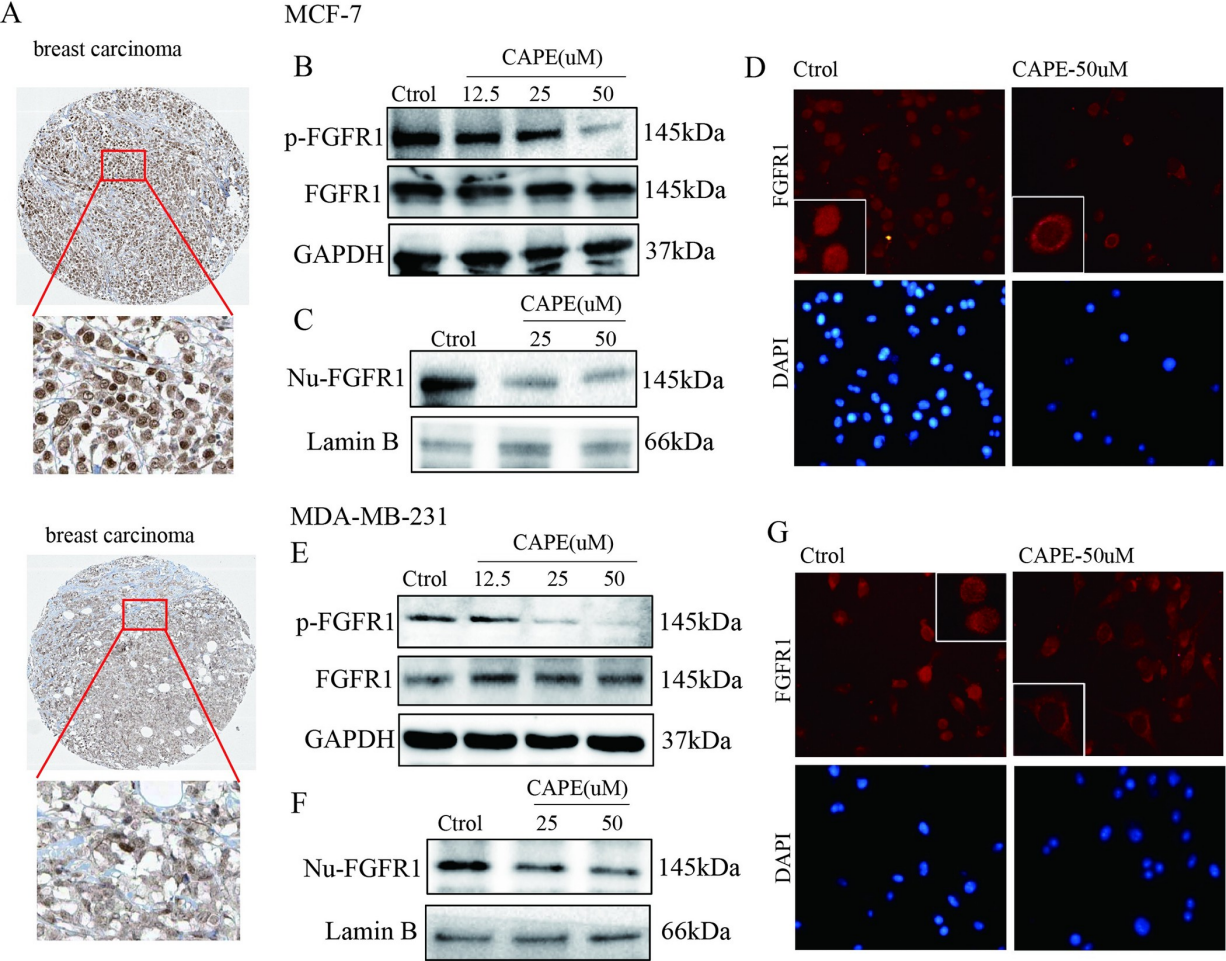
CAPE intercepted FGFR1 activation and nuclear transfer of MCF-7 and MDA-MB-231 cells. FGFR1 immunohistochemical staining of breast cancer patients from the human protein alts database (**A**). Cells were dosed with or without CAPE for 2 h, (**B, E**) expression of p-FGFR1 and FGFR1 were assessed by western blot. Cells were dosed with or without CAPE for 4 h, (**C, F**) nuclei protein was extracted for FGFR1 detection by westen blot and (**D, G**) immunofluorescence was performed for FGFR1 staining (400x). Experiments were repeated for 3 times.

FGFR1 is activated and transferred into the nucleus to promote metastasis of breast cancer. Immunohistochemical data of breast cancer tissue obtained from *the human protein alts database (https://www.proteinatlas.org/ENSG00000077782-FGFR1/pathology/breast+cancer)* (Fig 3A) shown that FGFR1 was markedly located in the nucleus. Further, we investigated the effect of CAPE on FGFR1. MCF-7 and MDA-MB-231 cells were incubated with CAPE at 12.5, 25 and 50 μM for 2h and then analyzed for FGFR1. As shown by western blot (Fig 3B and 3E), CAPE downregulated the expression of p-FGFR1 while the total proteins were no change. Subsequently, we examined FGFR1 nuclear translocation. MCF-7 and MDA-MB-231 cells were treated with CAPE for 4h, and results showed that CAPE reduced nuclear FGFR1 accumulation (Fig 3C, 3D, 3F and 3G). These findings demonstrated that CAPE inhibited FGFR1 activation and nuclear transfer.

### Overexpression of FGFR1 reversed the anti-tumor effect of CAPE

**Fig 4:**
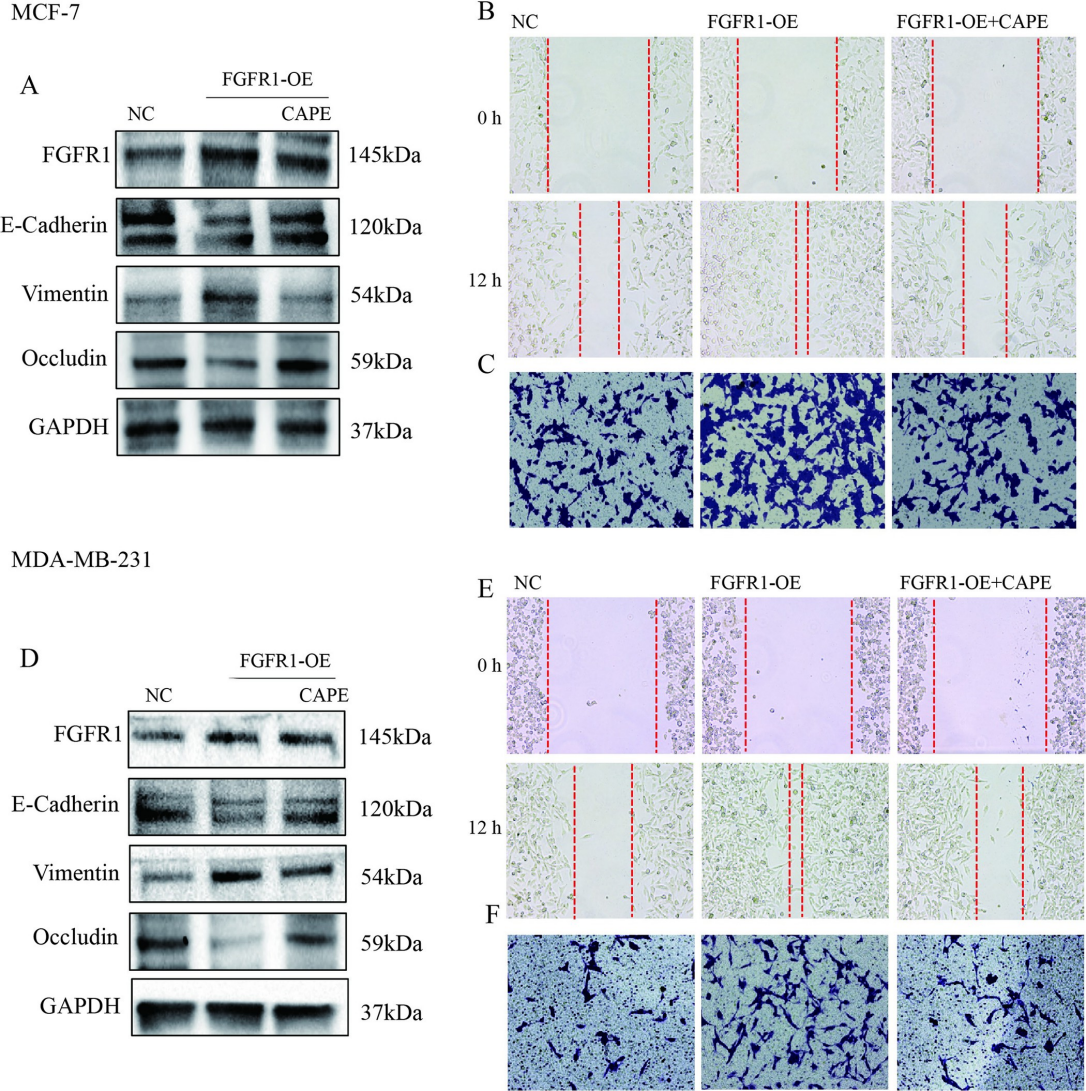
FGFR1 overexpression blocked the antitumor effect of CAPE in MCF-7 and MDA-MB-231 cells. (**A, D**) Cells were overexpressed with FGFR1, and then treated with CAPE. (**A, D**) Western blot was performed for E-cadherin, Vimentin and Occludin detection. (**B-C, D-E**) Wound healing and Transwell assay were used for migration (100x) and invasion (200x) evaluation. Experiments were repeated for 3 times.

To further clarify the role of FGFR1 in the antitumor metastasis effect of CAPE, MCF-7, and MDA-MB-231 cells were overexpressed with FGFR1, and then treated with CAPE 50 μM for 24h. Western blot showed that FGFR1 overexpression significantly up-regulates Vimentin and down-regulates E-cadherin and Occludin, promoting cell EMT transformation (Fig 4A and 4D). However, these EMT changes can be reversed by CAPE. In addition, wound healing and transwell experiments also showed the same results. The gap width in the wound healing experiment was narrower and the invasion cells in the transwell assay was more in the FGFR1 overexpression group while these were inhibited with CAPE treatment for 12h (Fig 4B, 4C, 4E and 4F). These results demonstrated that FGFR1 played an important role in CAPE’s antitumor effect.

### CAPE inactivated FGFR1 through MD2/FGFR1 complex inhibition

**Fig 5:**
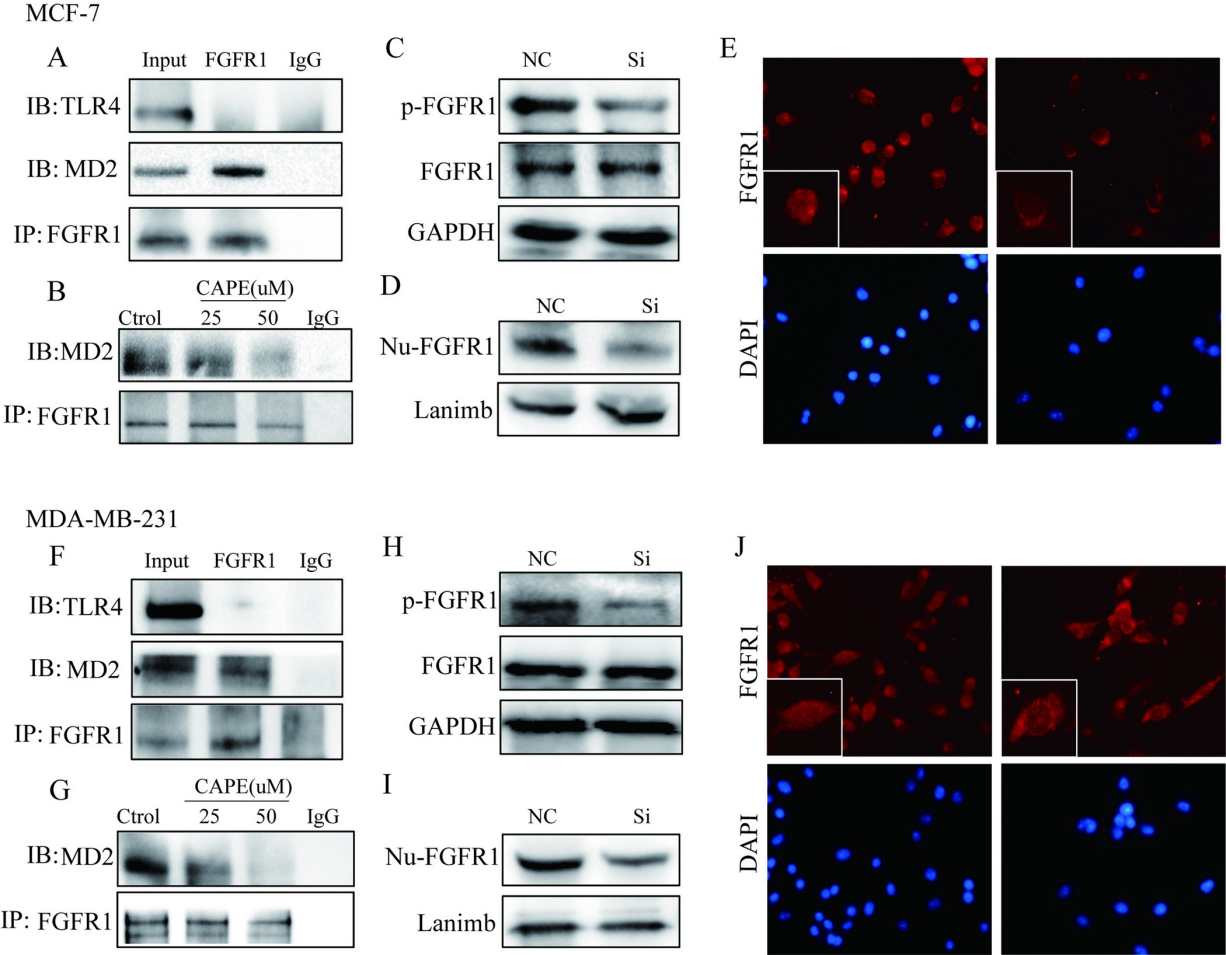
FGFR1 was combined with MD2, and silent MD2 suppressed FGFR1 activation and nuclear transfer in MCF-7 and MDA-MB-231 cells. (**A, F**) Western blot analyzed the immunoprecipitation of MD2 using FGFR1 antibody. (**B, G**) Cells were dosed with or without CAPE for 2 h, western blot analyzed the immunoprecipitation of MD2 using FGFR1 antibody. (**C, H**) Expression of p-FGFR1 and FGFR1 were determined in NC- or siMD2 cells by western blot. (**D-E, I-J**) Level of FGFR1 in the nucleus of NC- or siMD2 cells was determined by western blot and immunofluorescence. Experiments were repeated for 3 times.

MD2/TLR4 has been reported as a binding target of CAPE. Co-immunoprecipitation (Fig 5A and 5E) results showed that FGFR1 could combine with MD2 rather than TLR4 in MCF-7 and MDA-MB-231 cells. Besides, results (Fig 5B and 5F) showed the amount of FGFR1/MD2 complex was reduced after being treated with CAPE at 50 μM for 30min. Subsequently, we investigated the role of MD2 in the FGFR1 signal using siRNA targeting MD2. The silencing efficiency of siRNA targeting MD2 was detected and sequence 1 was chosen for the next experiments (S1 Fig). And as shown by western blot, MD2 silencing conspicuously inhibited the phosphorylation of FGFR1 (Fig 5C and 5G) in MCF-7 and MDA-MB-231 cells. In addition, MD2 silencing decreased the level of nuclear FGFR1 (Fig 5D and 5H). These results showed that MD2 may play a key role in CAPE-mediated FGFR1 signals inactivation.

### Silent MD2 suppressed cell migration and invasion

**Fig 6:**
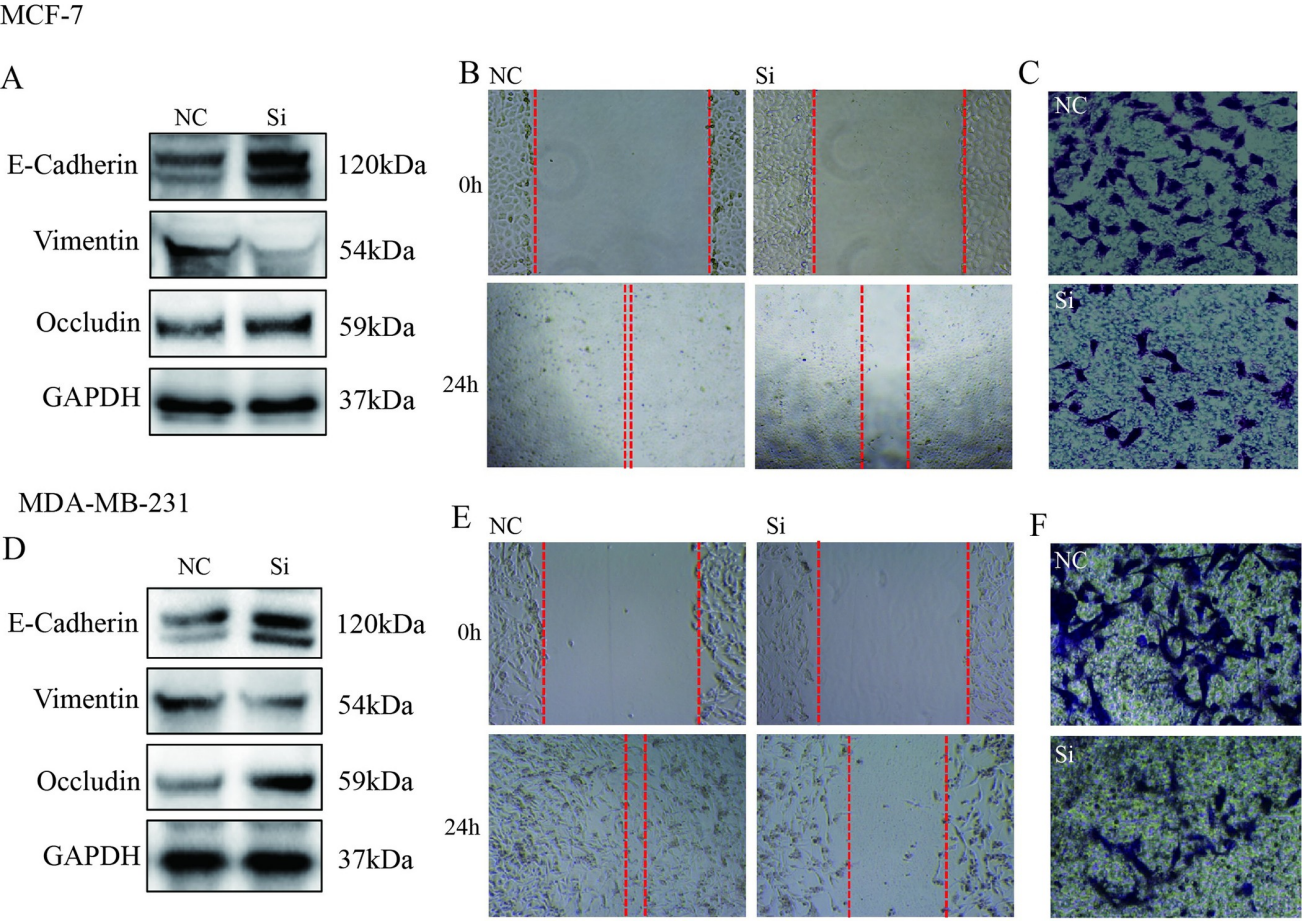
Silent MD2 inhibited the epithelial-mesenchymal transition, migration and invasion of MCF-7 and MDA-MB-231 cells. Cells were transfected with siRNA of MD2, (**A, D**) western blot was performed for E-cadherin, Vimentin and Occludin detection, (**B, E**) wound healing was used for migration evaluation(100x) and (**C, F**) Transwell assay was conducted for invasion evaluation (200x). Experiments were repeated for 3 times.

To further confirm the effect of MD2 inhibition on breast cancer metastasis, we transfected cells with siRNA targeting MD2 for 24h. MD2 silencing significantly changed expression of EMT-related indicators by down-regulated the expression of Vimentin and up-regulated the expression of E-cadherin and Occludin in protein levels (Fig 6A and 6D). Next, wound healing and transwell assays shown that MD2 silencing reduced the ability of cell migration and invasion with wider spacing (Fig 6B and 6E) and less invasive cells (Fig 6C and 6F). These data declared that MD2 was a target of CAPE to treat breast cancer metastasis.

## Discussion

Metastases occur in more than 90% of breast cancers, including lung metastases, bone metastases, and brain metastases, which are the main cause of death in breast cancer. At present, there is a lack of effective treatment for metastatic breast cancer. Chemotherapy is still the main method, but the adverse reaction is more, which seriously affects the quality of life of patients. Therefore, the discovery of effective therapeutic agents with low toxicity has become of great interest. CAPE is a natural product extracted from propolis and has strong pharmacological activity. Reports indicate that CAPE has a lethal effect on tumor cells with almost no toxicity [8,9]. Here, we examined the effect of CAPE on breast cancer cell metastasis and explored its mechanism.

Tumor metastasis is a series of complex metastatic cascades, including acquisition movement and invasion, angiogenesis, circulation survival, and colonization in metastatic sites [14]. Epithelial interstitial transformation (EMT) is a highly dynamic cellular process and is thought to be the starting point of metastasis of tumor cells. EMT causes changes in cell adhesion and polarity and results in increased motility and invasion [14]. EMT-associated proteins, E-cadherin and Occludin, act as epithelial markers and are believed to be inhibitors of invasion and growth of epithelial origin cancer types [15]. Vimentin is another important participant in EMT, assisting various signaling pathways to promote metastasis in response to dynamic structural changes in extracellular stimulations [16]. In this study, we found that CAPE could inhibit the migration and invasion of breast cancer cells. CAPE demonstrated dose-dependent inhibition on the EMT process through up-regulating E-cadherin and Occludin while down-regulating Vimentin.

FGFR family includes multiple subtypes, and the most common ones are FGFR1, FGFR2, FGFR3, and FGFR4. FGFRs play a variety of roles in normal physiological and developmental processes, such as embryonic organ development, tissue repair, angiogenesis, and metabolism [17,18]. Substantial evidence suggests that abnormal FGFR activation is involved in the pathogenesis of cancer [19–21]. FGFR2 is considered to be a susceptibility gene for breast cancer, FGFR4 is a driving factor for breast cancer, and FGFR1 overexpression is found in nearly 50% of breast cancer patients. Currently, FGFR1 activation has been shown to lead to increased cell proliferation, survival, and invasion both in mouse and human breast cancer cells [22,23]. Therefore, the effect of CAPE on FGFR1 signals was investigated in this study, and it was found that CAPE inhibited the phosphorylation of FGFR1 in a dose-dependent manner. In addition, FGFR1 nuclear transfer is another manifestation of its abnormal activation, and it has been confirmed that full-length FGFR1 in the nucleus can affect gene transcription [13]. In this study, we found that CAPE reduced the nuclear content of FGFR1 and showed a nuclear transfer inhibition of FGFR1 in breast cancer cells.

MD2, the ligand of TLR4, which plays an important role in the host immune system is a key target for the anti-inflammatory effects of CAPE [24,25]. Currently, some studies pointed out that there is a regulatory relationship between TLR4 and FGFR1. The inhibitor of TLR4, TAK-242, can inhibit the formation of FGFR1/TLR4/NLRP3 inflammasomes, and FGFR1 inhibitor can inhibit the LPS-induced hepatocyte inflammation through TLR4 [26,27]. However, there is no study on the role between MD2 and FGFR1. In this study, it was first found that FGFR1 could combine with MD2 instead of TLR4. There is a direct binding interaction between FGFR1 and MD2 in breast cancer cells. And, we also found CAPE could inhibit FGFR1 binding to MD2, while silencing MD2 inhibited activation and nuclear transfer of FGFR1 which meant MD2 is the membrane binding protein of CAPE inhibition on FGFR1. Besides, silencing MD2 blocked the EMT process, and inhibited the migration and invasion of breast cancer cells. Thus, CAPE suppressed breast cancer metastasis through inactivation of FGFR1 via MD2. Further study needs to prove this mechanism.

## Conclusion

Abnormal FGFR1 activation plays an important role in the regulation of breast cancer metastasis. Our study showed that CAPE could inhibit the phosphorylation of FGFR1, restrain FGFR1 nuclear transfer, and eventually block the EMT process as well as the migration and invasion of breast cancer cells. MD2 has been reported to be a membrane-binding target of CAPE. When we explored the mechanism of CAPE in regulating FGFR1 signal, we found that FGFR1 can form a complex with MD2 while CAPE reduced the amount of this complex. CAPE is a promising drug for inhibiting the metastasis of breast cancer cells.

## Supporting information

S1 Fig Silencing efficiency of siRNA targeting MD2. Western blot was performed to assess silencing efficiency.
(TIF)

S1 Raw images Raw western blot scans. (PDF)

S1 Table siRNA sequences of MD2. (DOCX)

S2 Table Primer sequences. (DOCX)
