# Supplementary figures and images for: Caffeic acid phenethyl ester suppresses metastasis of breast cancer cells by inactivating FGFR1 via MD2
Source: PLoS One. 2023 Jul 25;18(7):e0289031. doi: 10.1371/journal.pone.0289031 (PMC10368285; doi:10.1371/journal.pone.0289031)

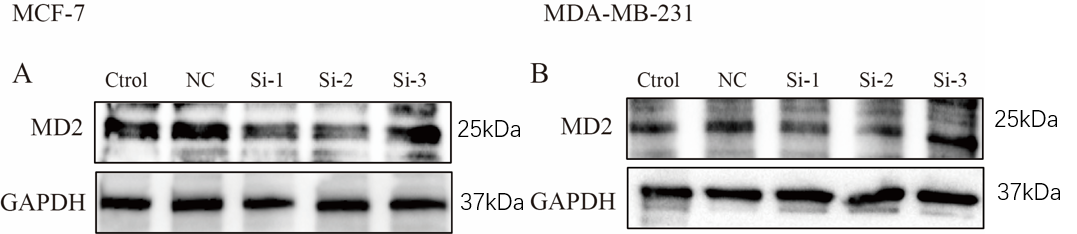

Supplement: S1 Fig — Western blot was performed to assess silencing efficiency. (TIF) [file pone.0289031.s001.tif]
