# Supplementary material for: Caffeic acid phenethyl ester suppresses metastasis of breast cancer cells by inactivating FGFR1 via MD2
Source: PLoS One. 2023 Jul 25;18(7):e0289031. doi: 10.1371/journal.pone.0289031 (PMC10368285; doi:10.1371/journal.pone.0289031)

Figure2

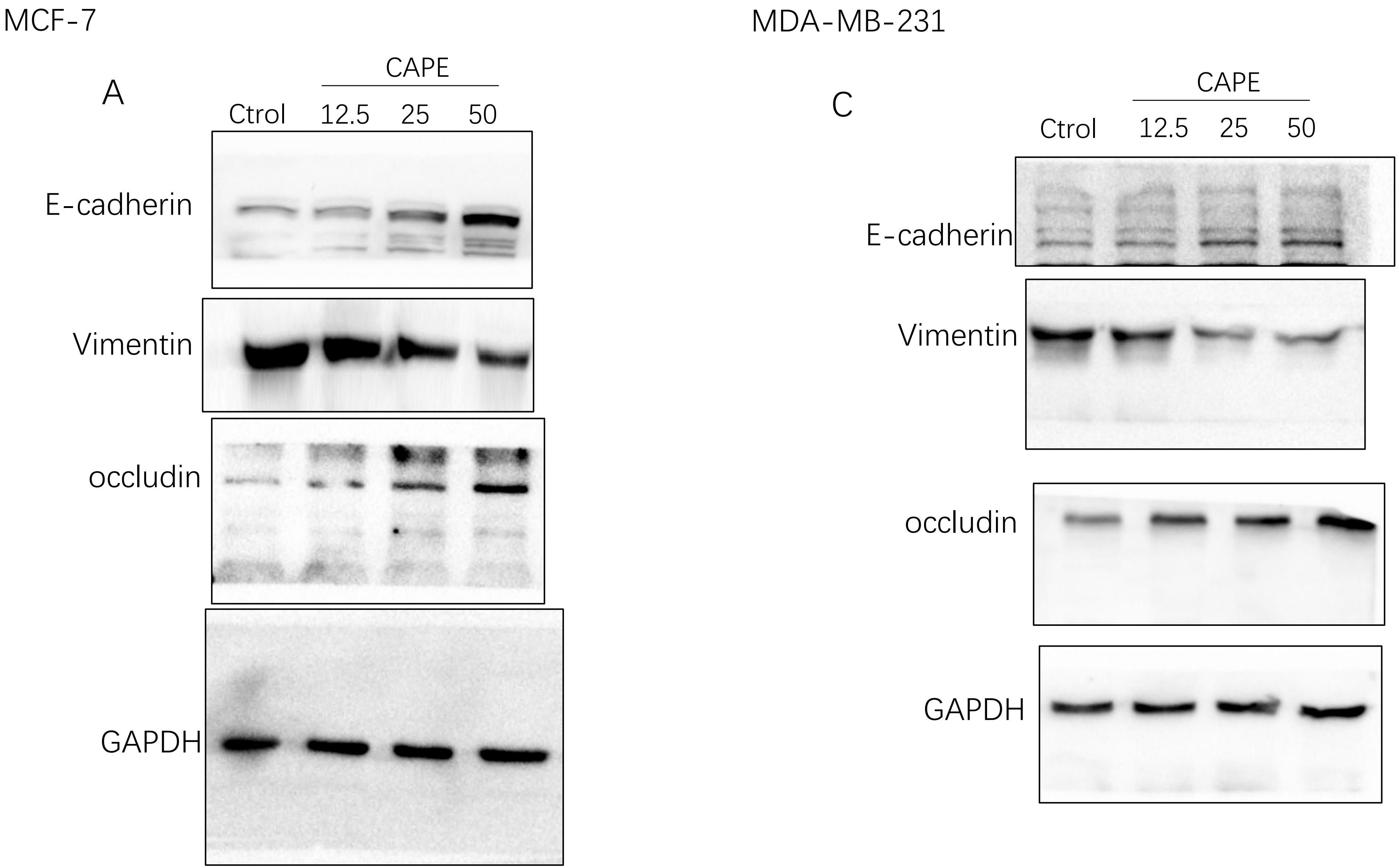
MCF-7
MDA-MB-231
A
C
CAPE
Ctrol
12.5
25
50
E-cadherin
Vimentin
occludin
GAPDH

Figure3

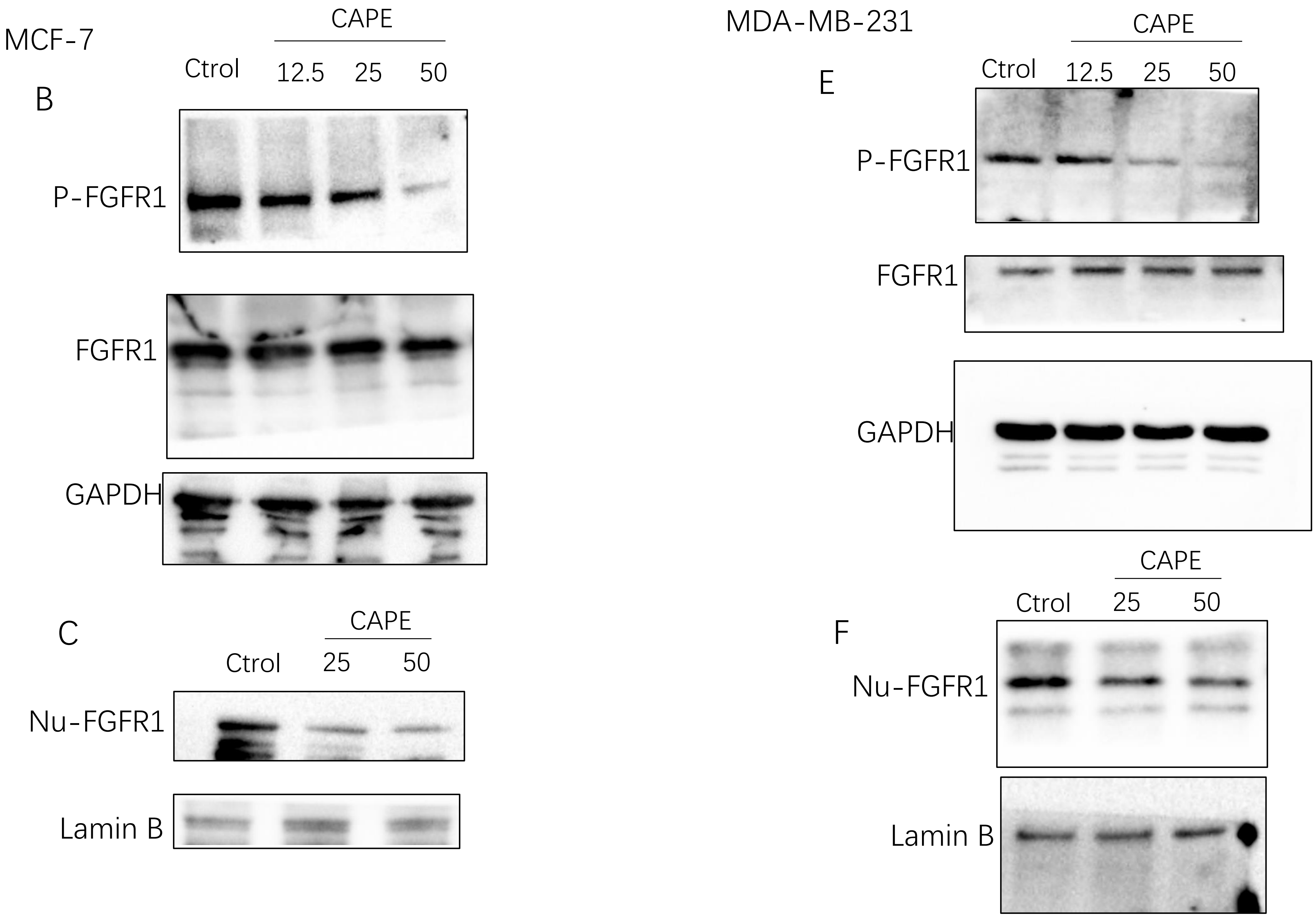
MCF-7
CAPE
Ctrol
12.5
25
50
B
P-FGFR1
FGFR1
GAPDH
C
CAPE
Ctrol
25
50
Nu-FGFR1
Lamin B
MDA-MB-231
CAPE
Ctrol
12.5
25
50
E
P-FGFR1
FGFR1
GAPDH
CAPE
Ctrol
25
50
F
Nu-FGFR1
Lamin B

Figure4

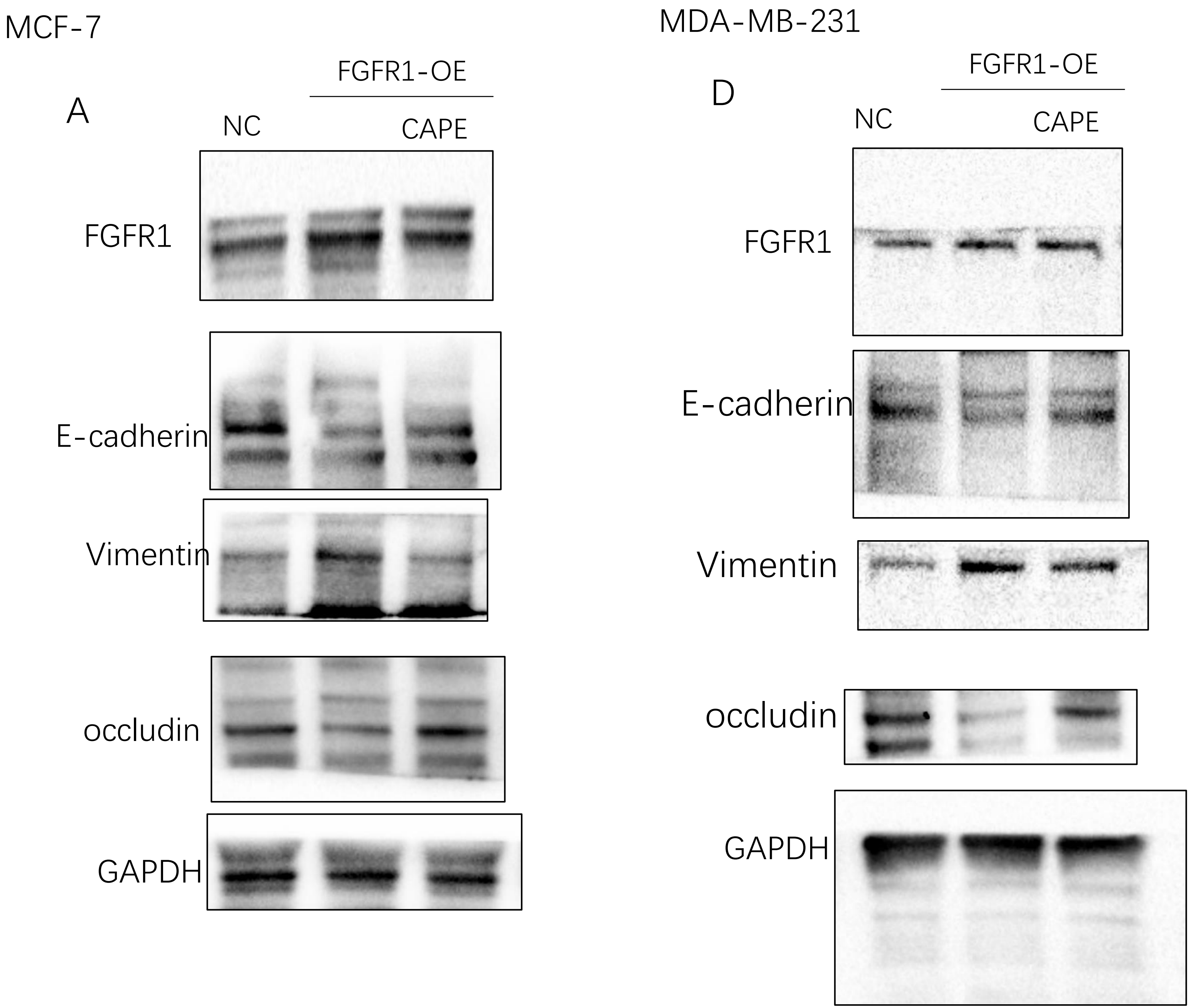
MCF-7
MDA-MB-231
A
FGFR1-OE
NC
CAPE
FGFR1
E-cadherin
Vimentin
occludin
GAPDH
D
FGFR1-OE
NC
CAPE
FGFR1
E-cadherin
Vimentin
occludin
GAPDH

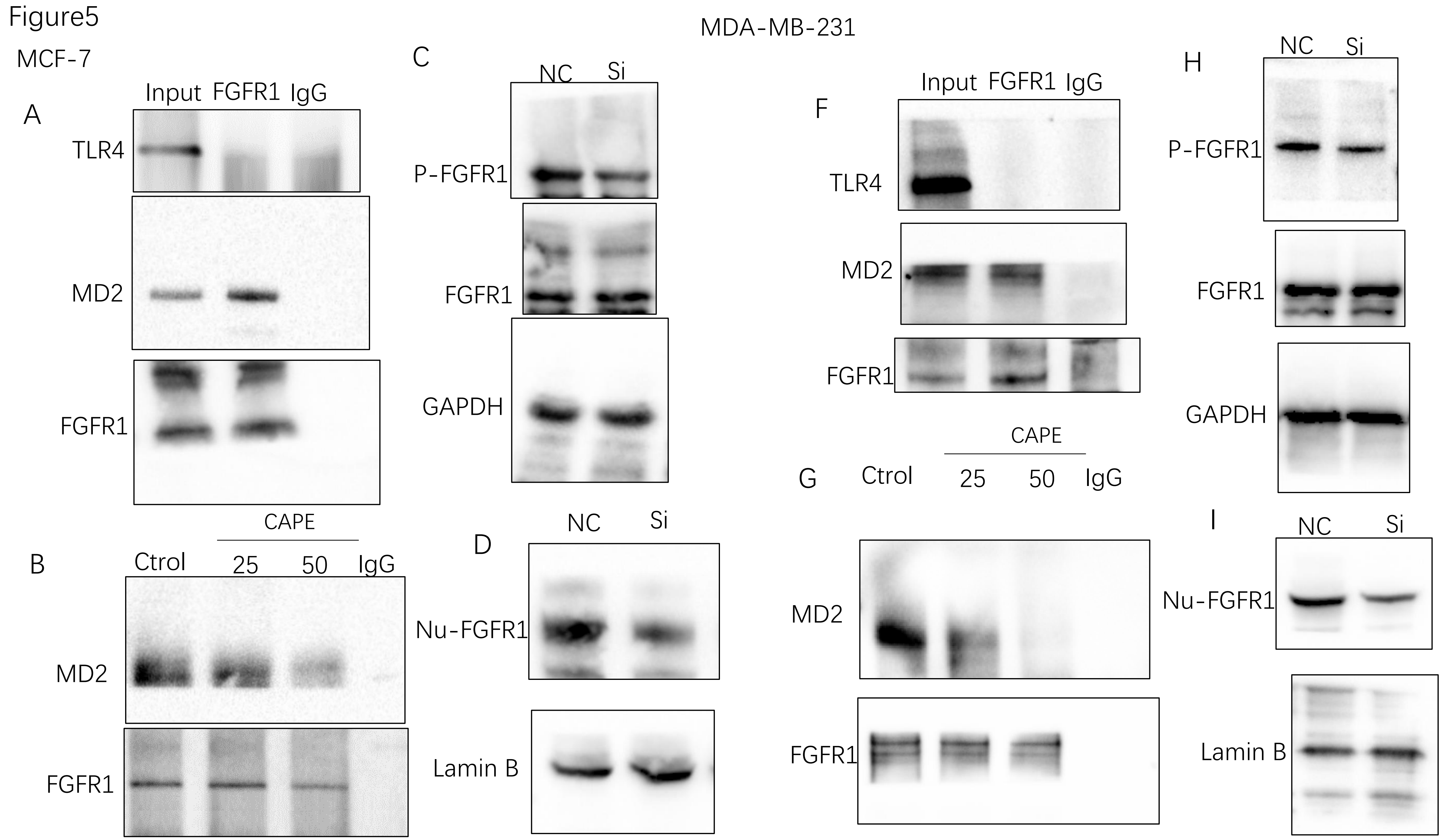
Figure5
MCF-7
MDA-MB-231
A
Input FGFR1 IgG
TLR4
MD2
FGFR1
B
CAPE
Ctrol 25 50 IgG
MD2
FGFR1
C
NC Si
P-FGFR1
FGFR1
GAPDH
D
NC Si
Nu-FGFR1
Lamin B
F
Input FGFR1 IgG
TLR4
MD2
FGFR1
G
CAPE
Ctrol 25 50 IgG
MD2
FGFR1
H
NC Si
P-FGFR1
FGFR1
GAPDH
I
NC Si
Nu-FGFR1
Lamin B

Figure6

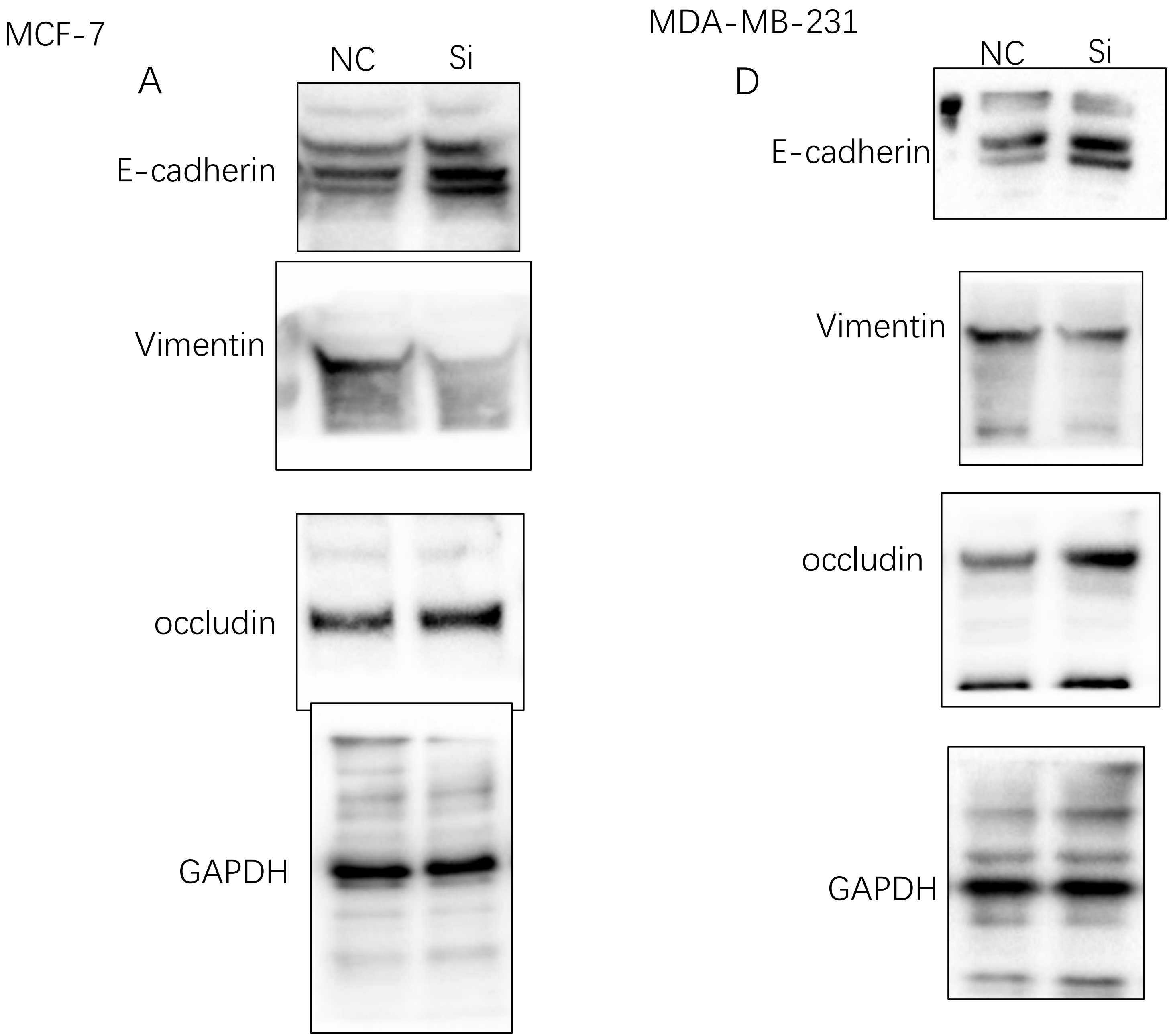
MCF-7
A
NC
Si
E-cadherin
Vimentin
occludin
GAPDH
MDA-MB-231
D
NC
Si
E-cadherin
Vimentin
occludin
GAPDH

Supplement Figure1

MCF-7

A

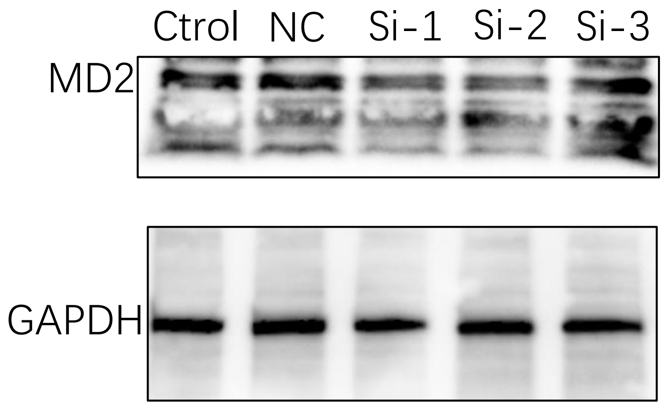


MDA-MB-231

B

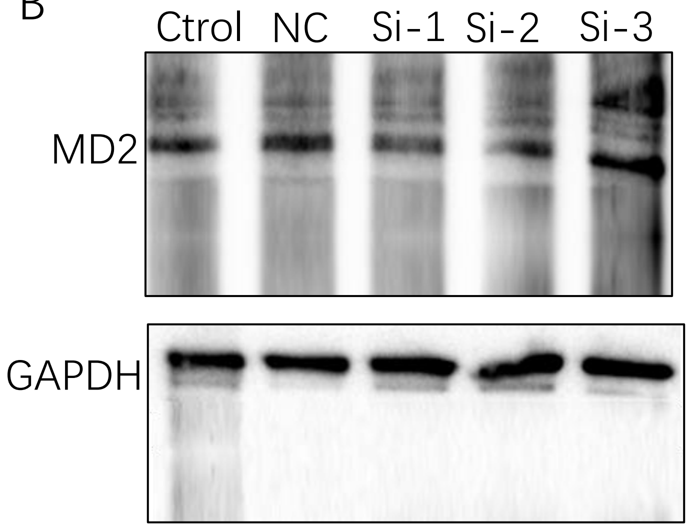

Supplement: S1 Raw images — (PDF) [file pone.0289031.s002.pdf]
