# Supplementary material for: Caffeic acid phenethyl ester suppresses metastasis of breast cancer cells by inactivating FGFR1 via MD2
Source: PLoS One. 2023 Jul 25;18(7):e0289031. doi: 10.1371/journal.pone.0289031 (PMC10368285; doi:10.1371/journal.pone.0289031)
Supplement: S1 Table — (DOCX) [file pone.0289031.s003.docx]

# S1 Table. siRNA sequences of MD2.

| **Name** | **Forward Primer** | **Reverse Primer** |
| --- | --- | --- |
| hs-MD2-si-1 | CAAGUAUUUCAUACACCUAdTdT | UAGGUGUAUGAAAUACUUGdTdT |
| hs-MD2-si-2 | GACUGUGAAUACAACAAUAdTdT | UAUUGUUGUAUUCACAGUCdTdT |
| hs-MD2-si-3 | GUUUGUCAUCCUACACCAAdTdT | UUGGUGUAGGAUGACAAACdTdT |
