# Supplementary material for: Caffeic acid phenethyl ester suppresses metastasis of breast cancer cells by inactivating FGFR1 via MD2
Source: PLoS One. 2023 Jul 25;18(7):e0289031. doi: 10.1371/journal.pone.0289031 (PMC10368285; doi:10.1371/journal.pone.0289031)
Supplement: S2 Table — (DOCX) [file pone.0289031.s004.docx]

# S2 Table. Primer sequences.

| **Name** | **Forward Primer** | **Reverse Primer** |
| --- | --- | --- |
| Vimentin | GACGCCATCAACACCGAGTT | CTTTGTCGTTGGTTAGCTGGT |
| Occludin | ACAAGCGGTTTTATCCAGAGTC | GTCATCCACAGGCGAAGTTAAT |
| GAPDH | TGTGGGCATCAATGGATTTGG | ACACCATGTATTCCGGGTCAAT |
| E-cadherin ATTTTTCCCTCGACACCCGAT TCCCAGGCGTAGACCAAGA | | |
